# Intriguing photo-control of exchange bias in BiFeO_3_/La_2/3_Sr_1/3_MnO_3_ thin films on SrTiO_3_ substrates

**DOI:** 10.1186/s11671-015-0824-4

**Published:** 2015-03-12

**Authors:** Kil Dong Sung, Tae Kwon Lee, Jong Hoon Jung

**Affiliations:** Department of Physics, Inha University, Incheon, 402-751 Republic of Korea

**Keywords:** 75.70.Ak, 75.47.Lx, 75.30.Et, BiFeO_3_/La_2/3_Sr_1/3_MnO_3_ thin film, Exchange bias, Photo-injection, Photo-conductivity

## Abstract

To date, electric fields have been widely used to control the magnetic properties of BiFeO_3_-based antiferromagnet/ferromagnet heterostructures through application of an exchange bias. To extend the applicability of exchange bias, however, an alternative mechanism to electric fields is required. Here, we report the photo-control of exchange bias in BiFeO_3_/La_2/3_Sr_1/3_MnO_3_ thin films on an SrTiO_3_ substrate. Through an *ex situ* pulsed laser deposition technique, we successfully synthesized epitaxial BiFeO_3_/La_2/3_Sr_1/3_MnO_3_ thin films on SrTiO_3_ substrates. By measuring magnetoresistance under light illumination, we investigated the effect of light illumination on resistance, exchange bias, and coercive field in BiFeO_3_/La_2/3_Sr_1/3_MnO_3_ thin films. After illumination of red and blue lights, the exchange bias was sharply reduced compared to that measured in the dark. With increasing light intensity, the exchange bias under red and blue lights initially decreased to zero and then appeared again. It is possible to reasonably explain these behaviors by considering photo-injection from SrTiO_3_ and the photo-conductivity of La_2/3_Sr_1/3_MnO_3_. This study may provide a fundamental understanding of the mechanism underlying photo-controlled exchange bias, which is significant for the development of new functional spintronic devices.

## Background

Within the last decade, the control of magnetic properties of materials using electric fields, and vice versa, has received great attention, as these effects may be utilized in a range of emerging spintronic device applications such as electric-field controlled tunneling magnetoresistance memory [1-4]. One of the strongest candidate materials for such functionality is BiFeO_3_ (BFO) because it exhibits coupling between ferroelectricity and antiferromagnetism at room temperature [5]. A promising application is the use of BFO as an antiferromagnetic layer for initiating exchange bias, a phenomenon that has been observed in antiferromagnetic/ferromagnetic multilayers [6]. Establishing an exchange bias in BFO/ferromagnet multilayers should facilitate the facile control of magnetism through electric fields [7-9].

There have been numerous studies of exchange bias in BFO-based multilayers, such as BFO/CoFe and BFO/CoFeB [7,10]. The exchange bias in BFO/ferromagnetic-metal heterostructures is strongly related to the ferroelectric domain walls of BFO, i.e., a 109° domain wall contributes to exchange bias, whereas 71° and 180° domain walls contribute to coercive field. On the other hand, the exchange bias in BFO/La_2/3_Sr_1/3_MnO_3_ (BFO/LSMO) is related to the hybridization of interfacial Fe and Mn orbitals rather than the BFO domain walls [11]. We previously investigated exchange bias control in BFO/LSMO through illumination with light rather than by manipulating the applied electric field [12]. This effect was attributed to a reduction in the hole doping ratio of LSMO and weakened exchange coupling between Fe and Mn spins at the interface caused by photo-injected electrons from the SrTiO_3_ (STO) substrate. However, further investigation for the mechanism underlying photo-controlled exchange bias is needed to extend its scope.

In this paper, we investigated the wavelength and intensity dependence of exchange bias in BFO/LSMO thin films on STO substrates. Upon illumination with red and blue lights, resistance increased whereas the exchange bias and coercive field decreased. With the increase of light intensity, the exchange bias for red light becomes zero at 0.1 mW/cm^2^ and for blue light at 8.81 μW/cm^2^, i.e., much smaller intensity for blue light than red light. In contrast, the coercive field for both red and blue lights decreased with almost the same manner. The mechanism of photo-controlled exchange bias for BFO/LSMO thin films on STO substrates is discussed in conjunction with the effect of photo-injection from STO and photo-conductivity of LSMO for unpinned and pinned spins in BFO.

## Methods

High-quality BFO/LSMO thin films were grown on an STO (001) substrate using pulsed laser deposition. A Q-switched 4ω Nd:YAG laser (266 nm, 5 Hz) was focused on BFO and LSMO ceramic targets with a fluence of approximately 1.5 J/cm^2^. Initially, we deposited an LSMO thin film at 750°C with an oxygen partial pressure (*P*_O2_) of 300 mTorr. A thin BFO film was then deposited *ex situ* on the LSMO/STO at 670°C and *P*_O2_ = 50 mTorr. Before BFO deposition, we blocked two regions of the LSMO using other STO substrates.

**Figure 1:**
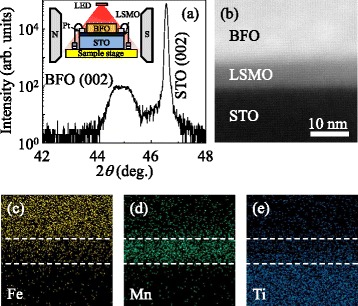
**Epitaxial growth of BiFeO**_**3**_ **/La**_**2/3**_ **Sr**_**1/3**_ **MnO**_**3**_ **thin film. (a)** High-resolution X-ray diffraction (HR-XRD) and **(b)** cross section scanning transmission electron microscopy (STEM) image of BiFeO_3_/La_2/3_Sr_1/3_MnO_3_ (BFO/LSMO) thin films on SrTiO_3_ (STO) substrates. Spatial mapping of **(c)** Fe, **(d)** Mn, and **(e)** Ti elements. The inset of (a) shows a schematic illustration of the magnetoresistance measurement for the BFO/LSMO thin film under red and blue light illumination.

The crystalline structure of the BFO/LSMO thin film was characterized using high-resolution X-ray diffraction (HR-XRD) (Bruker, AXS D8 Discover; Bruker AXS, Inc., Madison, WI, USA) and scanning transmission electron microscopy (STEM) with a Cs corrector (JEOL, 200 keV, JEM-2100 F; JEOL Ltd., Akishima-shi, Japan). The magnetic hysteresis curves of BFO/LSMO were obtained using the vibrating sample magnetometry functionality of a physical property measurement system (PPMS; Quantum Design, Inc., San Diego, CA, USA). For magnetoresistance measurement, we deposited Pt on the exposed LSMO regions and used a closed cycle cryostat (model 22, CTI-Cryogenics; Gardner Cryogenics, Bethlehem, PA, USA) equipped with an electromagnet (EM4-HVA, LakeShore Equipment Company, Carson, CA, USA). Thin films were illuminated through the optical window of a closed cycle cryostat with red (*λ* = 630 nm) and blue (*λ* = 460 nm) lights using light-emitting diodes (see the inset of Figure 1a).

## Results and discussion

Figure 1a, b shows the HR-XRD and cross section STEM results for BFO/LSMO thin films on STO substrates, respectively. From HR-XRD, it is apparent that the only a BFO (002) peak is observable near an STO (002). In addition, from STEM results, the lattice fringes for BFO, LSMO, and STO were clearly discernible without any noticeable lattice mismatches. Due to its thinness (approximately 5 nm), LSMO layer was only observable through STEM measurement. To further characterize the BFO/LSMO thin film on STO substrate, spatial mapping of the chemical elements Fe, Mn, and Ti is given in Figure 1c, d, e, respectively. While there are some ambiguities in our measurements, the majority of Fe, Mn, and Ti concentrations are observable at the top and bottom of the film and at the substrate, respectively. These results clearly suggest that BFO/LSMO multilayers should be epitaxially grown on an STO substrate using an *ex situ* pulsed laser deposition technique.

**Figure 2:**
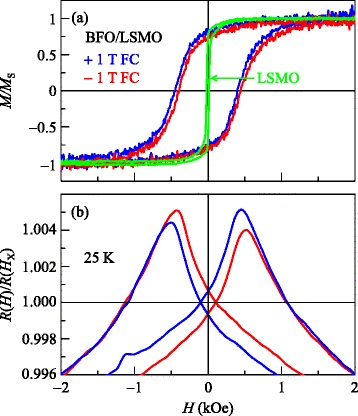
**Magnetization and magnetoresistance of BFO/LSMO thin film in the dark.** Normalized **(a)** magnetization *M/M*_S_ and **(b)** magnetoresistance *R*(*H*)/*R*(*H*_X_) curves of BFO/LSMO thin film after ±1 T field cooling (FC) at 25 K in under dark conditions. In (a), we show the normalized magnetization curve of LSMO thin film for comparison.

Figure 2a shows the magnetic hysteresis curves, *M*(*H*), for BFO/LSMO thin films at 25 K. We normalized the magnetic hysteresis curve to the saturated magnetization *M*_S_, i.e., *M*/*M*_S_. Before obtaining the *M*(*H*) curves for BFO/LSMO, we subtracted the contribution from diamagnetic STO substrates. One notable characteristic of the *M*(*H*) measurements is that the coercive field of BFO/LSMO was greatly enhanced compared to that of LSMO. In addition, the *M*(*H*) curves for BFO/LSMO shifted in the opposite direction to the applied magnetic field. It is worth noting that *M*(*H*) for LSMO did not shift in the direction of the applied magnetic field. These results clearly suggest that exchange bias is formed between antiferromagnet BFO and ferromagnet LSMO. To quantify the exchange bias, we defined the exchange bias field (*H*_E_) and coercive field (*H*_C_) as (*H*_C1_ + *H*_C2_)/2 and (*H*_C1_ – *H*_C2_)/2, respectively, where *H*_C1_ (*H*_C2_) represents the positive (negative) coercive field [6,13,14]. From the *M*(*H*) curve, we obtained *H*_E_ = −24.5 Oe (+22.8 Oe) and *H*_C_ = 425 Oe (420 Oe) for +1 T (−1 T) field cooling (FC).

Interestingly, the coercive fields observed in the *M*(*H*) curve coincided with peak positions in the magnetoresistance curve *R*(*H*) [8,9], as shown in Figure 2b. We normalized the magnetoresistance to the value of resistance at which the two curves coincided at *R*(*H*_X_), i.e., *R*(*H*)/*R*(*H*_X_). Similar to the *M*(*H*) curve, the *R*(*H*) curves showed a shift in the opposite direction to the applied magnetic field. The values of *H*_E_ and *H*_C_ obtained from the *R*(*H*) curve (*H*_E_ = −23 Oe and *H*_C_ = 480 Oe for +1 T) were close to those obtained from the *M*(*H*) curve (*H*_E_ = −24.5 Oe and *H*_C_ = 425 Oe for +1 T). This implies that it is possible to investigate the exchange bias of BFO/LSMO multilayers by measuring the magnetoresistance *R*(*H*) instead of the magnetic hysteresis curve *M*(*H*).

**Figure 3:**
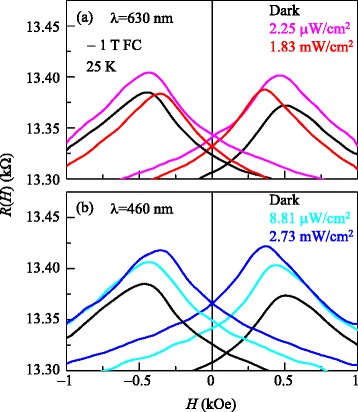
**Magnetoresistance of BFO/LSMO thin film under the illumination of light.** Magnetoresistance curves *R*(*H*) of BFO/LSMO thin films at 25 K after −1 T FC under light illumination at **(a)** *λ* = 630 and **(b)** *λ* = 460 nm. For comparison, *R*(*H*) curves under dark conditions are superposed in both (a) and (b).

After confirming the validity of magnetoresistance for the investigation of exchange bias, we illuminated the entire BFO/LSMO thin film surface with light at 25 K. Figure 3a, b shows the *R*(*H*) curves for BFO/LSMO thin films on an STO substrate after −1 T FC with light illumination at *λ* = 630 and 460 nm, respectively. For comparison, we superposed the *R*(*H*) curves obtained without any illumination. The *R*(*H*) curves under dark conditions, as shown in Figure 3a, b, are slightly different because we measured *R*(*H*) just before illumination of each wavelength after FC from 300 to 25 K to remove training effects [6].

Under illumination, the *R*(*H*) curves shifted upward and became symmetric, regardless of light wavelength, or intensity. However, detailed *R*(*H*) behaviors differed for each wavelength and intensity. For *λ* = 630 nm, exchange bias decreased with increasing intensity; *H*_E_ was estimated to be approximately 16.0 and approximately 5 Oe for light intensities of 2.25 μW/cm^2^ and 1.83 mW/cm^2^, respectively. In contrast, for *λ* = 460 nm, exchange bias initially decreased and then increased with increasing intensity. Specifically, *H*_E_ was estimated to be approximately 3 and 12 Oe for light intensities of 8.81 μW/cm^2^ and 2.73 mW/cm^2^, respectively.

**Figure 4:**
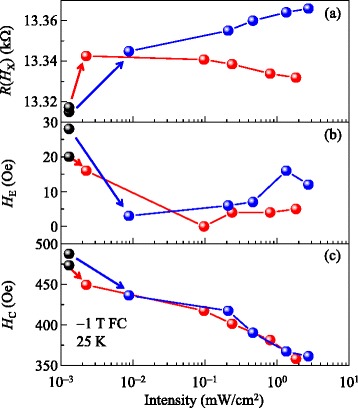
**Intensity and wavelength dependent resistance, exchange bias, and coercive field of BFO/LSMO thin film.** Light intensity dependence of **(a)** resistance *R*(*H*_X_), **(b)** exchange bias field *H*_E_, and **(c)** coercive field *H*_C_ for *λ* = 630 nm (red spheres) and 460 nm (blue spheres). In (a), (b), and (c), corresponding values under dark conditions (black spheres) are superposed for comparison.

To further investigate exchange bias, *R*(*H*) was measured under different light intensities. Figure 4a, b, c shows light intensity-dependent resistance *R*(*H*_X_), exchange bias field *H*_E_, and coercive field *H*_C_, respectively, for wavelengths of *λ* = 630 nm (red spheres) and 460 nm (blue spheres). For comparison, *R*(*H*_X_), *H*_E_, and *H*_C_ without illumination are superposed (black spheres). As the intensity of light increases, the resistance slightly decreased under red light, while increasing for blue light (Figure 4a). Exchange bias decreased down to almost zero and then became nearly constant for red light and slightly increased for blue light (Figure 4b). In contrast, the coercive field for both red and blue light decreased.

Two effects were considered to explain these behaviors: photo-injection from STO and photo-conductivity of LSMO, both of which modify carrier concentrations in LSMO [15-17]. When photo-injection from STO occurs, the electron concentration in LSMO should increase. When photo-conductivity occurs in LMSO, however, the hole concentration in LSMO should increase.

In the case of photo-injection from STO, both *λ* = 630 and 460 nm lights can excite electrons due to the oxygen vacancy and transfer them into the LSMO layer. The band gap of STO is 3.27 eV (*λ* = 379 nm) [18], and hence, photo-injection should be more significant for *λ* = 460 nm than for 630 nm. In addition, the intensity dependence of photo-injection should be more significant for *λ* = 460 nm than for 630 nm. In the case of photo-conductivity of LSMO, irradiation at both *λ* = 630 and 460 nm should increase hole concentration. Considering the metallic property of LSMO [19], the photo-conductivity effect should be nearly identical at both wavelengths. In addition, the dependence of photo-conductivity on light intensity should be nearly identical for both *λ* = 460 and 630 nm. Holes are the majority carrier in LSMO [19]; as the light intensity increases, therefore, the resistance will decrease for *λ* = 630 nm and increase for *λ* = 460 nm, consistent with Figure 4a.

This change in carrier concentration should affect the magnetic properties of LSMO. According to recent theories [11,20,21], super-exchange ferromagnetic coupling occurs in interfacial Fe and Mn ions, while super-exchange antiferromagnetic coupling occurs in interfacial Mn and neighboring Mn ions at the BFO/LSMO interface. In addition, electron (hole) doping induces ferromagnetic (antiferromagentic) coupling between interfacial Mn and its neighboring Mn ions due to competition between double-exchange and super-exchange couplings. Thus, photo-injected electrons from STO should induce modulation of interfacial Mn spins to align parallel to neighboring Mn spins, while photoconductivity-induced holes in LSMO should induce interfacial Mn spins antiparallel to neighboring Mn spins. Therefore, exchange bias should decrease with increasing electron concentration, consistent with the fact that *H*_E_ becomes nearly zero at approximately 0.1 mW/cm^2^ for red light and approximately 8.81 μW/cm^2^ for blue light (Figure 4b). Further increase of light intensity might result in the changes of compensated spins into uncompensated ones and/or pinned spins into unpinned ones due to the significantly increased electron and hole concentrations. While more investigation is required, such changes should result in the complicated behaviors of *H*_E_ with respect to wavelength, shown in Figure 4b.

Coercive field decreases with increasing intensity, regardless of wavelength. Note that, exchange bias and coercive field are related to pinned and unpinned spins of antiferromagnet, respectively [6]. We can observe the independence of coercive field with respect to wavelength from the fact that unpinned spins of BFO should be easily rotatable with respect to the exchange-coupled spins of LSMO. The carrier concentration in LSMO may be different for red and blue lights, which results in different angle rotation for LSMO spins. Due to exchange coupling between Mn and Fe spins, unpinned spins of BFO should also be rotated through the same angle as in LSMO. When we rotate the spin of LSMO through the applied magnetic field, the unpinned spin in BFO rotates accordingly. Therefore, the coercive field should have no wavelength dependence. We can observe the dependence of coercive field with respect to intensity from the fact that an increase in intensity should result in a corresponding increase of rotated spins of LSMO. Through exchange coupling between Mn and Fe spins, the number of rotated unpinned spins of BFO should also increase. When we rotate the spin of LSMO through the applied magnetic field, the increased number of unpinned BFO spins should be easily rotated, due to decreased spin drag effects [6]. Therefore, the coercive field should decrease as light intensity increases, consistent with Figure 4c.

## Conclusions

We reported intriguing photo-control of exchange bias in BiFeO_3_/La_2/3_Sr_1/3_MnO_3_ thin films on SrTiO_3_ substrates grown using an *ex situ* pulsed laser deposition technique. Using high-resolution X-ray diffraction and scanning transmission electron microscopy, we confirmed that the film was epitaxially grown without any significant lattice mismatches. After confirming that positive and negative coercive fields were the same, using magnetic hysteresis and magnetoresistance measurements, we investigated the change in exchange bias under light illumination. Upon illumination with red and blue lights, exchange bias decreased with increasing resistance. With increasing light intensity, exchange bias decreased to nearly zero for red light at much higher intensity than for blue light. In addition, the exchange bias appeared again for further increase of light intensity. Based on the relationship between wavelength, photo-injection from SrTiO_3_, and photo-conductivity of La_2/3_Sr_1/3_MnO_3_, we can reasonably explain changes in resistance, exchange bias, and coercive field. Competitive accumulation of holes and electrons at the interface is the key factor in determining exchange coupling between Fe and Mn spins and initiating exchange bias.
